# Effects of robotic and laparoscopic-assisted surgery on lymph node dissection and quality of life in the upper third of gastric cancer: A retrospective cohort study based on propensity score matching

**DOI:** 10.3389/fsurg.2022.1057496

**Published:** 2023-01-04

**Authors:** Jingxiao Fu, Yi Li, Xuechao Liu, Xuelong Jiao, Hongyu Qu, Yuhao Wang, Zhaojian Niu

**Affiliations:** Department of Gastroenterology, Affiliated Hospital of Qingdao University, Qingdao, China

**Keywords:** upper third gastric cancer, robotic surgery, laparoscopic surgery, lymph node dissection, quality of life

## Abstract

**Objective:**

The objective of this study was compare the effects of robot-assisted and laparoscopic-assisted surgery on lymph node dissection and quality of life in upper third gastric cancer patients undergoing radical total gastrectomy.

**Methods:**

The clinical and follow-up data of 409 patients with upper third gastric cancer who underwent total gastrectomy from July 2016 to May 2021 were enrolled. The patients were divided into a robotic group (*n* = 106) and a laparoscopic group (*n* = 303). Age, sex, body mass index, American Society of Anesthesiologists score, tumor size and location, pathological type, cT, cN, and cTNM were adjusted to offset selection bias. The patient characteristics, operative procedures, surgical outcomes, oncologic and pathologic outcomes, number of lymph node dissections, quality of life assessment, and nutritional status were compared between the two groups.

**Results:**

After propensity score matching, 61 cases were included in the robotic group and 122 cases were included in the laparoscopic group. The number of dissected lymph nodes (37.3 ± 13.5 vs. 32.8 ± 11.8, *P* = 0.022) significantly differed between the two groups. The number of lower mediastinal and subphrenic lymph nodes in the robotic group was greater than that in the laparoscopic group, and the difference was statistically significant (*P* < 0.001). Compared with the laparoscopic group, the total score of physical symptoms in the robotic group was significantly lower at 6 and 12 months after surgery (*P* = 0.03 and *P* = 0.001, respectively). The total social function score at 6 and 12 months after surgery was higher in the robotic group (*P* = 0.006 and *P* = 0.022). The quality of life scores were statistically significant only at 3 months after the operation (*P* = 0.047). A higher patient-generated subjective global assessment (PG-SGA) score is when the score significantly correlated (*P* < 0.001) with a higher related physical symptoms score, lower social function score, and lower quality of life score.

**Conclusion:**

Compared with laparoscopic radical gastrectomy, robotic radical gastrectomy is safe and feasible. Compared with laparoscopic radical gastrectomy, robotic radical gastrectomy was more refined, was associated with less surgical bleeding, and increased the quality of lymph node dissection. In addition, patients in the robotic group showed better postoperative quality of life.

## Introduction

In the past 40 years, the worldwide incidence rate of upper third gastric cancer (GC) has shown a significant upward trend (1, 2). Upper third gastric cancer is defined as adenocarcinoma of the upper third of the stomach, with or without esophagogastric junction adenocarcinoma, according to the Classification of the Japan Gastric Cancer Association (JGCA) (3). Although great progress has been made in targeting, immunological treatment, perioperative radiotherapy, and chemotherapy based on the molecular classification of upper third gastric cancer, surgery still plays an important role. Surgical methods, including traditional laparotomy and laparoscopy, represented by minimally invasive surgery and the Da Vinci robot, have become the main methods used to treat upper third gastric cancer cases worldwide. In 2002, Hashizume et al. carried out the world's first robot-assisted radical gastrectomy for gastric cancer (4). Robot-assisted surgery has a good 3D field of view, which is more suitable for narrow body cavity operations. The precise anatomy modified by the computer, the excellent suture technology under the microscope, and the movement of the 7-degree-of-freedom robot arm overcome the limits of the human body, which significantly reduced the dependence of the operator on the team. These unique advantages are unmatched by traditional laparoscopy. In addition, robotic surgery also has the advantages of a short learning curve, less surgical bleeding, an increased number of lymph node dissections, and other potential tumor control (5). However, few studies comparing laparoscopic and robotic lymph node dissection in upper third gastric cancer are currently available.

Radical gastrectomy combined with local lymph node dissection is the main treatment strategy for resectable gastric cancer (6). Especially for patients with advanced upper gastric cancer, radical total gastrectomy combined with D2 lymph node dissection is recommended. With the development of minimally invasive surgery and the improvement of postoperative quality of life (QOL), the recovery of postoperative somatic symptoms and social functions has gradually become the focus of attention for gastric cancer patients (7). The purpose of this study was to compare the effects of laparoscopic- and robotic-assisted gastrectomy combined with D2 lymph node dissection on the potential tumor control effect and quality of life of patients with upper third gastric cancer.

## Materials and methods

### Study design and participants

From July 2016 to May 2021, 409 patients with upper third gastric cancer consecutively received surgical treatment at the Gastrointestinal Surgery Department of Qingdao University Affiliated Hospital. Upper third gastric cancer is defined as adenocarcinoma of the upper third of the stomach, with or without esophagogastric junction adenocarcinoma, according to the Classification of the JGCA (3). The location of the primary carcinoma was determined by esophagogastroscopy.

Patients were divided into two groups based on whether they underwent robotic-assisted or laparoscopic-assisted surgery. Patients underwent propensity score matching analysis, and age, sex, body mass index (BMI), American Society of Anesthesiologists (ASA) score, tumor size, pathological type, cT, cN, and cTNM were adjusted to offset selection bias. The matched robot-assisted group was compared with the laparoscopic-assisted group based on preoperative basic information, perioperative complications, histopathological features, number of lymph nodes dissected during operation, postoperative quality of life, and nutritional status.

Preoperative tumor staging was assessed by computed tomography (CT) and gastroscopy. T stage and N stage were determined using the latest AJCC/UICC TNM staging system (8), and histological types were consistent with the Japanese classification of gastric cancer (3).

### Patients’ eligibility criteria

The patients’ eligibility criteria were as follows: (1) 12-month follow-up was completed and the follow-up data were complete; (2) gastric cancer in the upper third of the stomach; (3) first-time gastrectomy; (4) age >20 years for both sexes; (5) R0 gastrectomy; (6) no recurrence or distant metastasis; (7) performance status (PS) 0 or 1 based on the Eastern Cooperative Oncology Group scale; (8) sufficient capacity to understand and respond to the questionnaire; (9) no history of other diseases or operations that might influence the responses to the questionnaire; and (10) no organ failure or mental illness.

In addition, patients with a history of abdominal surgery, double primary tumors, and simultaneous resection of other organs were excluded.

### Surgery procedure

All patients had undergone curative resection and D1+/D2 lymphadenectomy in accordance with the Japanese guidelines for treating GC (3).

To obtain a better prognosis, a sufficient tumor edge should be ensured by the surgeons before specimen resection. If the tumor margins cannot meet the requirement and may be positive, intraoperative frozen pathology of the margin needs to be performed to exclude positive results. We stipulate performing uncut Roux-en-Y reconstruction after tumor resection.

The surgical procedure of robotic gastrectomy (RG) was very similar to that of laparoscopic gastrectomy (LG) in terms of trocar placement, surgical anatomical sequence, and anastomosis technique. The surgeons chose the extracorporeal method and stapling instrument methods for anastomosis to reinforce the hand-sewing according to the intraoperative conditions and extracorporeal anastomosis using a minilaparotomy.

### Perioperative management

The application of enhanced recovery after surgery (ERAS) has widely gained acceptance, and all patients were managed with the ERAS protocol during the perioperative period (9).

Before surgery, patients received education and exercise in lung function and prerehabilitation. For daily smokers and alcohol abusers, 1 month of abstinence was required before surgery. Chest, abdominal, and pelvic CT were performed to confirm the size and location of the tumor and imaging staging. Neoadjuvant chemotherapy was an option in cases with a large tumor or bulky lymph node metastasis. Cardiac ultrasound and pulmonary function tests were used to evaluate the tolerance of cardiopulmonary function for gastric cancer surgery. Lower extremity vascular ultrasound was used to evaluate the thrombus. Nutrition Risk Screening (NRS) 2002 was used to assess the nutritional status of patients: if the score was ≥3, the patient was given nutritional support (10). The correct evaluation of the patient's tolerance to surgery, reasonable treatment of other combined diseases, correction of anemia and water, and electrolyte disorders improved the patient's general condition.

On the day of surgery, the patients were allowed clear fluids for up to 2 h and solids for up to 6 h before the induction of anesthesia (11). A complex clear carbohydrate-rich drink designed for use within 2 h before anesthesia reduced hunger, thirst, anxiety and the length of stay, as well as postoperative insulin resistance.

After the surgery, the following measures were employed: (1) multimodal analgesia, including epidural analgesia and intravenous analgesia; (2) anasogastric tube, which should be removed on postoperative day 1 (POD1); (3) an abdominal drainage tube, which can be removed on POD3 when drainage fluid is clear and <100 ml/day, when the anastomotic status is good, or when no abdominal infection is found; (4) a urinary catheter, which should be removed on POD1; (5) venous thromboembolism (VTE) prevention, which included mechanical measures (intermittent pneumatic leg compression and elastic stockings) for patients with increased VTE risk; (6) oral feeding: we stipulated that patients can consume a clear fluid diet on POD1–2, semiliquid diet on POD3–4, and soft blended diet on POD5 if tolerable and then gradual transition to a normal diet based on the premise of patient's tolerance and no severe complication (including anastomosis leakage, ileus, high risk of gastroplegia, etc.); (7) movement: we encouraged early ambulation for 1 h/day on POD1 and prepared an appropriate scheme of movement for patients. The time of ambulation should properly increase to 4 h/day on POD7 based on the patient's status and need; (8) discharge: patients could be discharged from the hospital on POD7 according to the discharge criteria (without postoperative complications and primary disease that requires current intervention).

According to the postoperative pathological stage and Chinese Society of Clinical Oncology (CSCO) guidelines for the diagnosis and treatment of gastric cancer, patients with stage II/III gastric cancer were treated with 5-fluorouracil-based regimens, either XELOX or S-1, for six to eight cycles. The patients were followed up for 1 year and once at 3, 6, and 12 months. The follow-up included physical examination and laboratory examination. At each follow-up, the diet, physical symptoms, and social function recovery were collected by telephone contact. The deadline for follow-up was May 2022.

### Data collection and clinical analysis

Patients were enrolled in this study, and two data management staff members were assigned to collect relevant data. The basic characteristics of patients collected before surgery were age, sex, body mass index, ASA score, and hematologic indices (complete blood count, blood biochemistry, tumor biomarkers, etc.). The operation was characterized by estimated blood loss, operative time, and cost. Postoperative outcomes were mean maximum body temperature during the first 3 days, pain Numerical Assessment Scale (NAS) score on the first 3 days after surgery, days of bowel function recovery, time to start soft diet, complications, and adverse events. Morbidity was described based on the Clavien–Dindo classification of JOCG criteria for postoperative complications and according to the Common Terminology Criteria for Adverse Events (CTCAE 5.0) (12–15). The oncologic and pathologic outcomes were the number of lymph nodes removed, pathological proximal and distal margins, TNM stage, tumor size and location, Lauren classification, and tumor cell differentiation. After discharge, a 1-year follow-up, which included QOL questionnaires, periodic physicals, laboratory examinations, and abdominal CT every 3 months at the outpatient department, began on time.

Assessment of postoperative quality of life: The quality of life assessment scale was constructed with reference to the quality of life questionnaire-core 30 (QLQ-C30) (16, 17) and the gastric cancer specific module scale (quality of life questionnaire-stomach 22, qlq-sto22) (18, 19) designed by the European Organization for Research and Treatment of Cancer (EORTC) in Chinese. The new scale combines the advantages of the above two scales, mainly including physical symptom scores and social function evaluations. According to the scoring procedure of the EORTC scoring manual, the score is linearly converted into a score of 0–100. The higher the function score is, the better the function, and the higher the symptom score is, the more serious the symptoms (20). Quality of life score = (100 − score of physical symptom scale + score of social function scale)/2.

Nutritional parameters after gastrectomy were assessed on the basis of changes in serum prealbumin, albumin, hemoglobin, meal size and times, foods with different degrees of hardness and softness, prognostic nutritional index (PNI), and body weight at 3, 6, and 12 months after surgery (21). PNI was calculated using the following formula: 10 × serum albumin value (g/dl) + 0.005 × lymphocyte count in peripheral blood (22). On the CT images, the cross-sectional area of the psoas muscle was measured at the level of the third lumbar vertebra (L3). Psoas muscle index (PMI) = (area of the psoas muscle at L3 cm^2^)/(height m^2^). The 1-year change rate was calculated as follows: (nutrition-related indicators at one year after surgery − preoperative)/(preoperative × 100) (23, 24). Considering the relationship between nutritional status and quality of life, the patient-generated subjective global assessment (PG-SGA) score was used to assess the association with quality of life.

### Statistical analysis

All data were processed using SPSS 26.0 and R software (version 4.0.2). To eliminate the potential deviation caused by the lack of equal distribution between the two groups, a logistic regression model with the following covariates was used to calculate the propensity score: age, sex, body mass index, American Society of Anesthesiologists score, tumor location, tumor size, pathological type, cT, cN, and cTNM. Matching was performed at a ratio of 1:2, and a caliper width of 0.01 standard deviation was specified (25). Categorical variables are expressed as examples (%), and the chi-square or Fisher exact test was used. Continuous variables conforming to a normal distribution are expressed as X ± s, and a paired *t*-test was used for comparisons between groups. Nutrition-related indices were compared before the operation and at 1, 3, and 6 months after the operation, and repeated-measures ANOVA was used. *P* < 0.05 indicates that the difference is statistically significant.

### Ethics statement

The data for this study were collected in the course of general clinical practice, so informed consent signed by each patient was obtained for any surgical and clinical procedure. This protocol was in line with the ethical guidelines of the World Medical Association Declaration of Helsinki adopted by the 18th World Medical Association Congress held in Helsinki, Finland, in June 1964. Institutional Review Board approval was not needed. Since this study was retrospective, patients’ consent was not required for inclusion in the study.

## Results

### Patient characteristics

**Figure 1:**
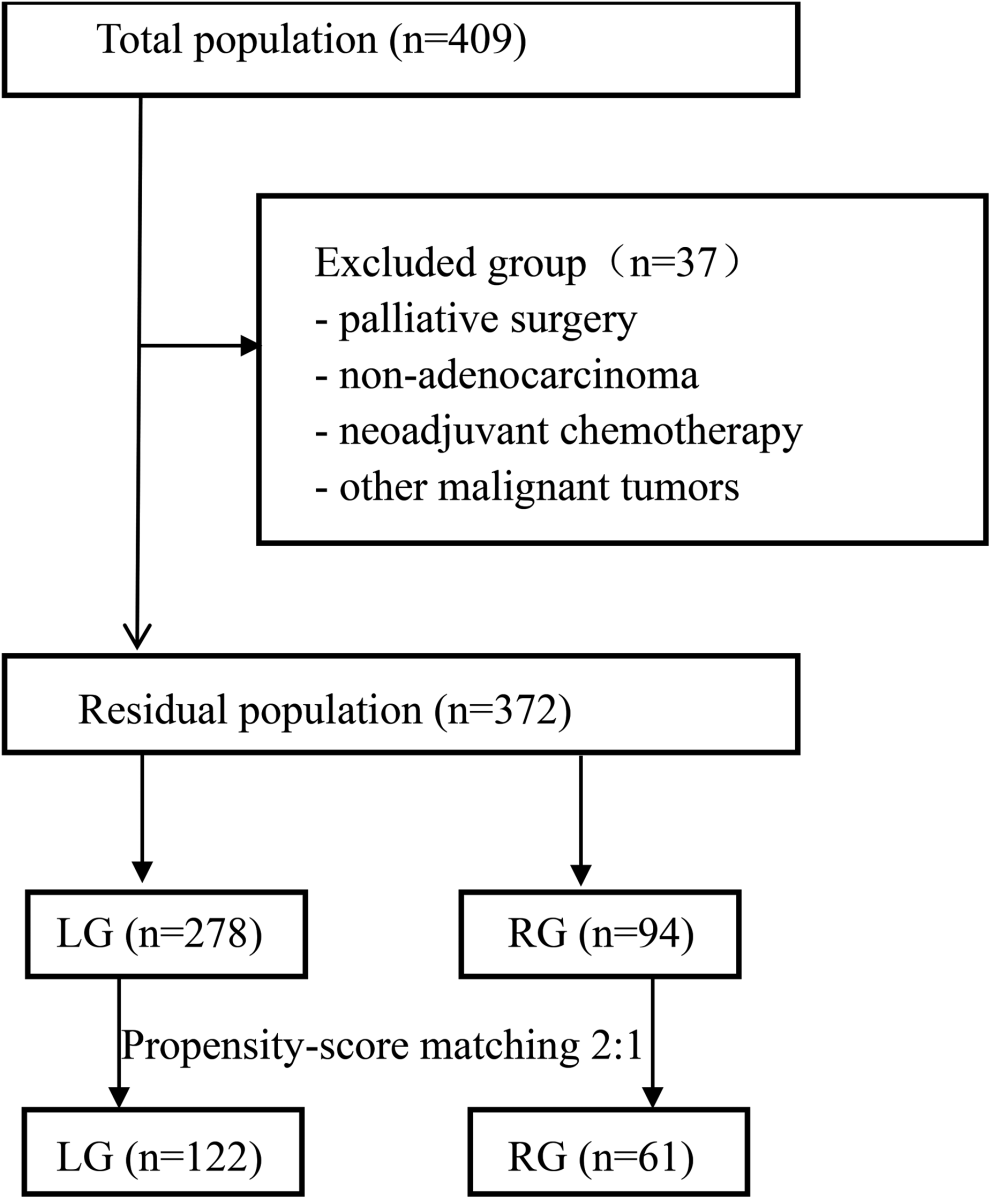
Selection schema of patients. LG, laparoscopic gastrectomy; RG, robotic gastrectomy.

A total of 409 upper third gastric cancer patients underwent surgery (Figure 1). Among these patients, 37 patients were excluded from the study due to palliative surgery (*n* = 7), non-adenocarcinoma (*n* = 4), complications with other malignant tumors (*n* = 6), neoadjuvant chemotherapy (*n* = 9), and loss to follow-up (*n* = 11). Finally, a total of 372 patients who underwent laparoscopic-assisted surgery (278 patients) or robotic-assisted surgery (94 patients) were enrolled in this study. After 1:2 matching between the RG group and LG group, 61 patients were included in the RG group and 122 patients were included in the LG group.

The basic clinical characteristics of the patients in the two groups are shown in Table 1. Age, sex, BMI, preoperative nutritional indicators, or comorbidities did not significantly differ between groups in the entire cohort. The matched baseline features were well balanced.

**Table 1:** Patient characteristics.

Factor	Entire cohort		*P*	Matched cohort		*P*
	RG (*n* = 106)	LG (*n* = 303)		RG (*n* = 61)	LG (*n* = 122)	
Age, year ± SD	63.34 ± 7.91	64.08 ± 8.52	0.434	64.11 ± 8.03	64.42 ± 8.12	0.807
Sex			0.773			0.412
Male, *n* (%)	74 (69.8)	216 (71.3)		42 (68.9)	91 (74.6)	
Female, *n* (%)	32 (30.2)	87 (28.7)		19 (31.1)	31 (25.4)	
BMI, kg/m^2^ ± SD	24.69 ± 4.01	25.15 ± 3.14	0.229	24.36 ± 4.12	25.01 ± 3.64	0.278
ASA physical status			0.808			0.909
0–1, *n* (%)	43 (40.6)	127 (41.9)		18 (29.5)	37 (30.3)	
≥2, *n* (%)	63 (59.4)	176 (58.1)		43 (70.5)	85 (69.7)	
Preoperative Hb, g/L ± SD	131.85 ± 12.01	130.41 ± 13.17	0.322	133.21 ± 13.41	133.76 ± 12.74	0.787
Preoperative albumin, g/L ± SD	41.21 ± 3.03	40.92 ± 2.78	0.367	42.17 ± 3.12	41.62 ± 2.61	0.210
Psoas muscle index, cm^2^/m^2^ ± SD	172.24 ± 29.12	171.26 ± 31.04	0.776	173.02 ± 31.95	171.27 ± 32.05	0.728
Lymphocyte count, 10^9^/L ± SD	1.39 ± 0.42	1.42 ± 0.35	0.472	1.38 ± 0.43	1.41 ± 0.36	0.620
Preoperative prealbumin, g/L ± SD	239.52 ± 31.22	241.67 ± 34.21	0.569	242.86 ± 33.14	240.14 ± 32.19	0.594
NRS 2002 score			0.275			0.916
<3, *n* (%)	69 (65.1)	179 (59.1)		36 (59.0)	71 (58.2)	
≥3, *n* (%)	37 (34.9)	124 (40.9)		25 (41.0)	51 (41.8)	
Her2			0.560			0.929
0, *n* (%)	71 (67.0)	215 (70.9)		43 (70.5)	87 (71.3)	
+, *n* (%)	23 (21.7)	59 (19.5)		11 (18.0)	23 (18.9)	
++, *n* (%)	3 (2.8)	13 (4.3)		2 (3.3)	5 (4.1)	
+++, *n* (%)	9 (8.5)	16 (5.3)		5 (8.2)	7 (5.7)	
History of smoking, *n* (%)	67 (63.2)	184 (60.7)	0.652	33 (54.1)	69 (56.6)	0.752
FEV1.0, % ± SD	76.3 ± 8.7	75.4 ± 8.2	0.339	77.8 ± 9.5	76.3 ± 9.4	0.312
Number of comorbidities			0.612			0.991
0, *n* (%)	51 (48.1)	137 (45.2)		24 (39.3)	46 (37.7)	
1, *n* (%)	37 (34.9)	126 (41.6)		29 (47.5)	61 (50.0)	
2, *n* (%)	13 (12.3)	29 (9.6)		6 (9.8)	11 (9.0)	
3, *n* (%)	5 (4.7)	11 (3.6)		2 (3.3)	4 (3.3)	
Comorbidities			0.987			0.954
Hypertension	31 (29.2)	112 (37.0)		22 (36.1)	47 (38.5)	
Diabetes	18 (17.0)	61 (20.1)		15 (24.6)	27 (22.1)	
Hepatic disease	3 (2.8)	7 (2.3)		2 (3.3)	3 (2.5)	
Cardiac disease	4 (3.8)	9 (3.0)		3 (4.9)	5 (4.1)	
Cerebrovascular disease	3 (2.8)	10 (3.3)		1 (1.6)	4 (3.3)	
Asthma	1 (0.9)	5 (1.7)		1 (1.6)	2 (1.6)	
History of pulmonary tuberculosis	1 (0.9)	4 (1.3)		0 (0)	1 (0.8)	

RG, robotic gastrectomy; LG, laparoscopic gastrectomy; BMI, body mass index; ASA, American Society of Anesthesiologists; NRS, Nutrition Risk Screening.

### Operative procedures and surgical outcomes

The operation time did not significantly differ between the two groups (*P* = 0.531). Compared with that in the laparoscopic group, the intraoperative blood loss in the robotic group was lower (45.7 ± 13.9 vs. 53.4 ± 21.6 ml, *P* = 0.012). In addition, the time to bowel function recovery (2.02 ± 1.21 vs. 2.63 ± 1.09, *P* = 0.001) and start of a soft diet (3.41 ± 1.64 vs. 4.07 ± 1.32, *P* = 0.004) in the robotic group were better than those in the laparoscopic group. The pain NAS score on the first postoperative day in the robotic group (1.51 ± 0.23 vs. 2.29 ± 0.28, *P* < 0.001) was lower than that in the laparoscopic group. No significant difference was detected in the highest temperature or complications. However, cost was higher in the robotic group than in the laparoscopic group (79,810.6 ± 7,126 vs. 63,102.1 ± 4,137, *P* < 0.001) (Table 2).

**Table 2:** Operative procedures and surgical outcomes.

Factor	Entire cohort		*P*	Matched cohort		*P*
	RG (*n* = 106)	LG (*n* = 303)		RG (*n* = 61)	LG (*n* = 122)	
Operation time (min ± SD)	182.2 ± 36.4	174.3 ± 37.1	0.059	176.1 ± 39.1	172.3 ± 38.3	0.531
Estimated blood loss (ml ± SD)	48.4 ± 11.5	55.1 ± 24.8	0.008	45.7 ± 13.9	53.4 ± 21.6	0.012
Lymph node dissection, *n* (%)			0.268			0.221
D1+	21 (19.8)	46 (15.2)		14 (23.0)	19 (15.6)	
D2	85 (80.2)	257 (84.8)		47 (77.0)	103 (84.4)	
Bowel function recovery (days ± SD)	2.49 ± 1.34	2.89 ± 1.16	0.004	2.02 ± 1.21	2.63 ± 1.09	0.001
Start of soft diet (days ± SD)	3.74 ± 1.51	4.13 ± 1.62	0.031	3.41 ± 1.64	4.07 ± 1.32	0.004
Pain numerical assessment scale score						
Postoperative day 1	1.62 ± 0.41	2.03 ± 0.37	<0.001	1.51 ± 0.23	2.29 ± 0.28	<0.001
Postoperative day 2	1.54 ± 0.29	1.59 ± 0.33	0.167	1.47 ± 0.26	1.52 ± 0.31	0.138
Postoperative day 3	1.24 ± 0.22	1.26 ± 0.27	0.493	1.21 ± 0.25	1.18 ± 0.21	0.230
Body temperature during the first 3 days^a^						
Postoperative day 1	37.5°C ± 1.3°C	37.3°C ± 1.7°C	0.271	37.6°C ± 1.6°C	37.4°C ± 1.3°C	0.366
Postoperative day 2	37.8°C ± 1.5°C	37.6°C ± 1.4°C	0.215	37.5°C ± 1.4°C	37.6°C ± 1.6°C	0.679
Postoperative day 3	37.3°C ± 1.2°C	37.2°C ± 1.3°C	0.487	37.2°C ± 1.3°C	37.3°C ± 1.2°C	0.606
Overall complication, *n* (%)			>0.999			>0.999
Anastomotic leakage	1 (0.9)	2 (0.6)		0 (0)	0 (0)	
Anastomotic stenosis	3 (2.8)	7 (2.3)		1 (1.6)	1 (0.8)	
Cholecystitis	1 (0.9)	4 (1.3)		0 (0)	0 (0)	
Pancreatitis	2 (1.9)	5 (1.7)		0 (0)	2 (1.6)	
Pancreatic fistula	1 (0.9)	5 (1.7)		0 (0)	1 (0.8)	
Intraperitoneal hemorrhage	1 (0.9)	4 (1.3)		1 (1.6)	2 (1.6)	
Fluid abscess	2 (1.9)	5 (1.7)		0 (0)	2 (1.6)	
Wound infection	2 (1.9)	3 (0.9)		0 (0)	1 (0.8)	
Wound dehiscence	1 (0.9)	3 (0.9)		0 (0)	1 (0.8)	
Pneumonia	4 (3.8)	11 (3.6)		2 (3.3)	5 (4.1)	
Chyle leakage	1 (0.9)	3 (0.9)		0 (0)	2 (1.6)	
Ileus	2 (1.9)	4 (1.3)		1 (1.6)	2 (1.6)	
Adverse events, *n* (%)			0.847			0.744
Anemia^b^	27 (25.5)	78 (25.7)		11 (18.0)	23 (18.9)	
Lymphocytopenia^c^	4 (3.8)	6 (2.0)		1 (1.6)	2 (1.6)	
Creatinine increased^d^	2 (1.9)	4 (1.3)		1 (1.6)	1 (0.8)	
Hypo-pre-albuminemia^e^	23 (21.7)	63 (20.8)		13 (21.3)	24 (19.7)	
Hyperbilirubinemia^f^	7 (6.6)	19 (6.3)		3 (4.9)	7 (5.7)	
AST/ALT increased^g^	5 (4.7)	13 (4.3)		3 (4.9)	6 (4.9)	
Hypernatremia^h^	0 (0)	1 (0.3)		0 (0)	0 (0)	
Hyponatremia^i^	6 (5.7)	21 (6.9)		4 (6.6)	10 (8.2)	
Hyperkalemia^j^	3 (2.8)	7 (2.3)		1 (1.6)	3 (2.5)	
Postoperative hospital stay (days ± SD)	6.9 ± 4.4	7.3 ± 5.2	0.479	6.6 ± 4.1	7.2 ± 4.8	0.405
30-day reoperation, *n* (%)	1 (0.9)	4 (3.8)	>0.999	0 (0)	1 (0.8)	>0.999
30-day readmission, *n* (%)	5 (4.7)	12 (4.0)	0.737	1 (1.6)	3 (2.5)	>0.999
Medical cost (dollars ± SD)	81,942.7 ± 8,796	65,917.2 ± 5,138	<0.001	79,810.6 ± 7,126	63,102.1 ± 4,137	<0.001

RG, robotic gastrectomy; LG, laparoscopic gastrectomy; ALT, alanine aminotransferase; AST, aspartate aminotransferase.

^a^ The highest body temperature.

^b^ Male patients Hb < 120 g/L, female patients Hb < 110 g/L.

^c^ Lymphocyte count <1.1 × 10^9^/L.

^d^ Creatinine > 132 μmol/L.

^e^ Pre-albumin <200 mg/L.

^f^ Total bilirubin >22 µmol/L.

^g^ AST/ALT > 2.

^h^ Na > 147 mmol/L.

^i^ Na < 137 mmol/L.

^j^ K > 5.3 mmol/L.

### Oncologic and pathologic outcomes

Tumor location and size, proximal and distal resection margins, histological type, or Lauren classification did not significantly differ between the two groups (*P* > 0.05). After propensity matching, no significant difference in pTNM staging was detected between the two groups (*P* > 0.05) (Table 3).

**Table 3:** Oncologic and pathologic outcomes.

Variable	Entire cohort		*P*	Matched cohort		*P*
	RG (*n* = 106)	LG (*n* = 303)		RG (*n* = 61)	G (*n* = 122)	
Tumor location, *n* (%)			0.854			0.919
EG junction	29 (27.4)	71 (23.4)		17 (27.9)	31 (25.4)	
Cardia	9 (8.5)	24 (7.9)		7 (11.5)	11 (9.0)	
Fundus	33 (31.1)	99 (32.7)		18 (29.5)	39 (32.0)	
Upper body	35 (33.0)	109 (36.0)		19 (31.1)	41 (33.6)	
Tumor size (cm ± SD)	4.1 ± 3.1	4.7 ± 2.9	0.073	4.2 ± 2.7	4.4 ± 3.2	0.676
Pathological proximal margin (cm ± SD)	2.3 ± 1.9	2.6 ± 2.1	0.195	2.1 ± 1.7	2.4 ± 1.5	0.224
Pathological distal margin (cm ± SD)	5.1 ± 1.7	5.3 ± 2.6	0.461	4.9 ± 1.4	5.2 ± 1.7	0.235
Histological type, *n* (%)			0.804			0.931
Poorly differentiated	64 (60.4)	197 (65.0)		34 (55.7)	73 (59.8)	
Moderately differentiated	31 (29.2)	82 (27.1)		21 (34.4)	40 (32.8)	
Well differentiated	7 (6.6)	16 (5.3)		4 (6.6)	6 (4.9)	
Undifferentiated	4 (3.8)	8 (2.6)		2 (1.9)	3 (2.5)	
Histology (Lauren classification), *n* (%)			0.848			0.989
Intestinal	30 (28.3)	87 (28.7)		17 (27.9)	36 (29.5)	
Diffuse	39 (36.8)	99 (32.7)		21 (34.4)	41 (33.6)	
Mixed	31 (29.2)	95 (31.4)		19 (31.1)	36 (29.5)	
Indeterminate	6 (5.7)	22 (7.3)		4 (6.6)	9 (7.4)	
T stage, *n* (%)			0.338			0.962
T1	20 (18.9)	42 (13.9)		7 (11.5)	15 (12.3)	
T2	16 (15.1)	33 (10.9)		12 (19.7)	27 (22.1)	
T3	58 (54.7)	189 (62.4)		37 (60.7)	69 (56.6)	
T4a	12 (11.3)	39 (12.9)		5 (8.2)	11 (9.0)	
N stage, *n* (%)			0.608			0.906
N0	38 (35.8)	97 (32.0)		23 (37.7)	42 (34.4)	
N1	59 (55.7)	185 (61.1)		33 (54.1)	69 (56.6)	
N2	9 (8.5)	21 (6.9)		5 (8.2)	11 (9.0)	
pTNM stage, *n* (%)			0.874			0.993
IA	17 (16.0)	44 (14.5)		9 (14.8)	21 (17.2)	
IB	14 (13.2)	30 (9.9)		7 (11.5)	16 (13.1)	
IIA	23 (21.7)	74 (24.4)		17 (27.9)	31 (25.4)	
IIB	34 (32.1)	104 (34.3)		19 (31.1)	36 (29.5)	
IIIA	13 (12.3)	32 (10.6)		7 (11.5)	13 (10.7)	
IIIB	5 (4.7)	19 (6.3)		2 (3.3)	5 (4.1)	

RG, robotic gastrectomy; LG, laparoscopic gastrectomy; EG, esophagogastric.

**Figure 2:**
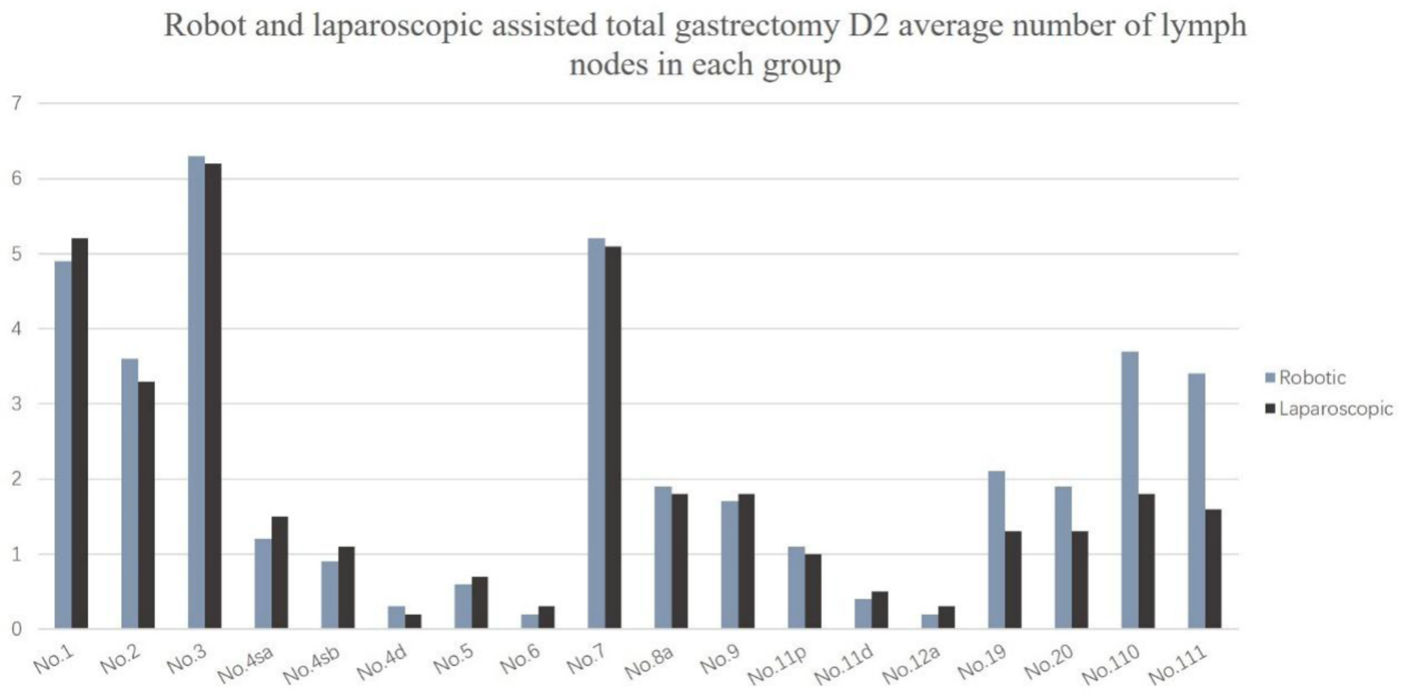
Robot and laparoscopic assisted total gastrectomy D2 average number of lymph nodes in each group.

The number of dissected lymph nodes (37.3 ± 13.5 vs. 32.8 ± 11.8, *P* = 0.022) was significantly different between the two groups. The number of lower mediastinal and subphrenic lymph nodes in the robotic group was greater than that in the laparoscopic group, and the difference was statistically significant (*P* < 0.001) (Table 4). The total number of abdominal lymph nodes and the number of abdominal lymph nodes at each station between the two groups were not statistically significant (*P* > 0.05) (Figure 2).

**Table 4:** Comparison of lymph node dissection between robot and laparoscopic group.

Lymph node dissection	RG (*n* = 61)	LG (*n* = 122)	*P*
Retrieved lymph nodes	37.3 ± 13.5	32.8 ± 11.8	0.022
Subphrenic lymph nodes			
No.19	2.4 ± 0.9	1.1 ± 0.7	<0.001
No.20	2.3 ± 0.6	1.2 ± 0.5	<0.001
Inferior mediastinal lymph nodes			
No.110	3.3 ± 1.6	1.7 ± 0.8	<0.001
No.111	3.2 ± 1.4	1.9 ± 1.3	<0.001
Laparoscopic lymph nodes	23.4 ± 11.2	24.6 ± 12.6	0.530

RG, robotic gastrectomy; LG, laparoscopic gastrectomy.

**Figure 3:**
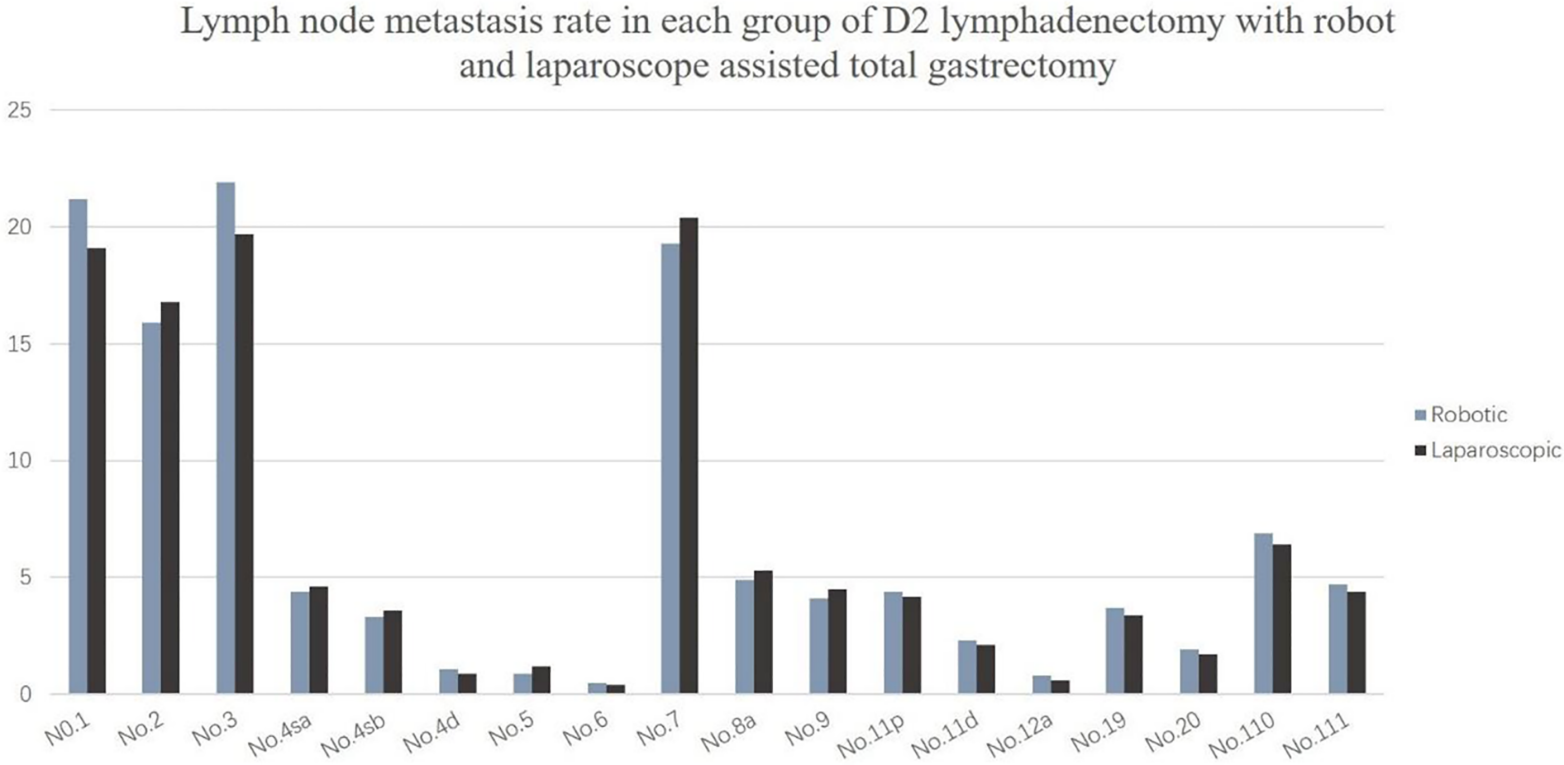
Lymph node metastasis rate in each group of D2 lymphadenectomy with robot and laparoscope assisted total gastrectomy.

The lymph node metastasis rates of No. 1, No. 2, No. 3, and No. 7 were the highest, all approximately 20%, followed by No. 8a, No. 9, No. 11p, and No. 110. The lymph node metastasis rate was close to 5%, and the lymph node metastasis probability of other stations was less than 5% (Figure 3).

### Quality of life assessment

In the robotic group, the total scores of physical symptoms before surgery and 3, 6, and 12 months after surgery were 7.9 ± 5.1, 16.4 ± 7.3, 9.6 ± 5.3, and 6.7 ± 1.9, respectively. Compared with the laparoscopic group, the total score of physical symptoms in the robotic group was significantly lower at 6 and 12 months after surgery (*P* = 0.03 and *P* = 0.001, respectively). In the robotic group, the total social function scores were 94.7 ± 7.3, 71.6 ± 12.7, 81.6 ± 8.4, and 90.3 ± 7.8 before surgery and 3, 6, and 12 months after surgery, respectively. In the laparoscopic group, the scores were 93.9 ± 7.1, 69.2 ± 9.6, 77.4 ± 10.2, and 87.1 ± 9.3, respectively. Compared with the laparoscopic group, the total score at 6 and 12 months after surgery was higher in the robotic group (*P* = 0.006 and *P* = 0.022). The quality of life scores of the robotic group were 95.2 ± 8.1, 78.0 ± 13.1, 85.7 ± 9.4, and 92.9 ± 7.8 before surgery and 3, 6, and 12 months after surgery, respectively. The scores of the laparoscopic group were 94.9 ± 8.9, 74.4 ± 10.6, 83.4 ± 10.5, and 90.7 ± 8.6, respectively. The difference between the two groups at each time point was statistically significant only at 3 months after the operation (*P* = 0.047) (Table 5).

**Table 5:** Assessment of quality of life.

Factor	RG (*n* = 61)	LG (*n* = 122)	*P*	RG (*n* = 61)	LG (*n* = 122)	*P*
	Preoperative			Three months after surgery		
Physical symptoms	6.9 ± 3.1	7.2 ± 3.7	0.587	16.4 ± 7.3	18.7 ± 9.3	0.093
Dysphagia	10.3 ± 6.3	10.9 ± 5.7	0.518	27.8 ± 12.3	30.9 ± 10.8	0.082
Sour regurgitation	9.2 ± 7.1	8.9 ± 6.5	0.776	10.3 ± 6.3	12.4 ± 7.1	0.052
Belching	5.1 ± 4.2	5.4 ± 3.9	0.633	11.2 ± 6.1	14.1 ± 8.7	0.021
Abdominal pain	8.1 ± 4.9	8.3 ± 4.1	0.771	15.7 ± 6.8	19.1 ± 7.3	0.003
Diarrhea	6.9 ± 5.1	7.1 ± 5.2	0.805	13.4 ± 7.1	16.6 ± 8.7	0.014
Fatigue	7.1 ± 4.7	7.3 ± 4.4	0.777	18.7 ± 10.3	21.2 ± 9.8	0.111
Anxious	3.1 ± 1.2	2.9 ± 1.5	0.366	15.7 ± 9.2	18.4 ± 8.3	0.047
Insomnia	3.2 ± 1.4	3.7 ± 1.8	0.059	13.6 ± 8.7	16.2 ± 11.1	0.111
Social function	94.7 ± 7.3	93.9 ± 7.1	0.477	71.6 ± 12.7	69.2 ± 9.6	0.155
Independent living	96.3 ± 14.1	95.1 ± 15.2	0.607	82.1 ± 17.2	77.6 ± 18.1	0.109
Hobby	89.1 ± 15.7	90.4 ± 16.1	0.604	78.3 ± 16.8	73.4 ± 17.1	0.068
Exercise	93.1 ± 16.2	91.9 ± 17.3	0.652	74.3 ± 13.2	69.2 ± 12.7	0.012
Work efficiency	84.9 ± 19.2	86.1 ± 10.1	0.580	72.5 ± 17.4	64.7 ± 16.2	0.003
Quality of life score	95.2 ± 8.1	94.9 ± 8.9	0.825	78.0 ± 13.1	74.4 ± 10.6	0.047
	Six months after surgery		* *	One year after surgery		* *
Physical symptoms	9.6 ± 5.3	11.4 ± 5.2	0.030	6.7 ± 1.9	7.8 ± 2.1	0.001
Dysphagia	16.4 ± 6.7	18.4 ± 9.7	0.150	12.3 ± 3.1	13.1 ± 2.9	0.087
Sour regurgitation	8.2 ± 4.3	8.6 ± 3.7	0.515	8.1 ± 3.3	8.3 ± 3.7	0.721
Belching	7.1 ± 3.9	9.4 ± 4.5	0.001	6.2 ± 2.2	6.7 ± 2.4	0.174
Abdominal pain	12.3 ± 4.9	14.4 ± 5.7	0.015	9.3 ± 3.6	10.6 ± 3.3	0.016
Diarrhea	12.9 ± 5.3	14.1 ± 4.9	0.130	9.9 ± 4.7	10.1 ± 4.6	0.783
Fatigue	11.3 ± 5.2	13.7 ± 6.5	0.013	8.4 ± 3.9	9.2 ± 3.1	0.134
Anxious	12.4 ± 3.3	14.2 ± 3.5	0.001	8.3 ± 2.6	10.4 ± 2.9	0.013
Insomnia	10.2 ± 3.7	11.8 ± 4.0	0.010	7.7 ± 2.4	8.6 ± 2.3	0.015
Social function	81.6 ± 8.4	77.4 ± 10.2	0.006	90.3 ± 7.8	87.1 ± 9.3	0.022
Independent living	87.6 ± 15.1	84.1 ± 13.7	0.117	91.4 ± 13.7	88.3 ± 11.6	0.111
Hobby	82.3 ± 14.2	77.6 ± 12.1	0.021	86.5 ± 12.9	83.7 ± 15.6	0.228
Exercise	83.7 ± 17.3	78.9 ± 14.7	0.051	88.3 ± 15.3	83.2 ± 14.4	0.028
Work efficiency	77.9 ± 16.2	72.1 ± 12.4	0.008	82.7 ± 13.6	78.1 ± 12.7	0.025
Quality of life score	85.7 ± 9.4	83.4 ± 10.5	0.150	92.9 ± 7.8	91.7 ± 8.6	0.360

RG, robotic gastrectomy; LG, laparoscopic gastrectomy.

### Nutritional status

The preoperative baseline data of the two groups were very balanced (Tables 6, 7). Although body weight did not significantly differ between the two groups during the same period after surgery, the 1-year change rate was statistically significant [(−8.1 ± 1.7) vs. (−8.7 ± 1.9), *P* = 0.039]. PMI significantly differed between the two groups at 3 and 6 months after the operation (*P* < 0.05), but no significant difference was detected in the 1-year change rate after the operation (*P* > 0.05). The proportion of meal size change [(−16.4 ± 3.9)% vs. (−18.1 ± 4.3)%, *P* = 0.001] and the proportion of meal time change [(29.3 ± 6.5)% vs. (31.2 ± 7.1)%, *P* = 0.081] significantly differed between groups. At 3, 6, and 12 months after the operation, the proportion of solid diet in the robotic group was higher than that in the laparoscopic group, but this difference was not significant (*P* > 0.05). As seen in Table 8, a higher PG-SGA score significantly correlated (*P* < 0.001) with a higher related physical symptom score, lower social function score, and lower quality of life score.

**Table 6:** Postoperative recovery of nutrition-related indicators in two groups.

Factor	RG (*n* = 61)	LG (*n* = 122)	*P*	RG (*n* = 61)	LG (*n* = 122)	*P*
	Preoperative			Three months after surgery		
Body weight (kg)	63.2 ± 9.7	62.9 ± 9.4	0.841	57.8 ± 6.5	56.1 ± 6.1	0.084
Psoas muscle index (cm^2^/m^2^)	15.2 ± 2.1	15.4 ± 2.3	0.569	10.5 ± 1.8	9.8 ± 1.6	0.008
Serum albumin (g/L)	42.7 ± 5.6	43.1 ± 6.2	0.672	37.5 ± 4.7	37.9 ± 4.3	0.566
Serum prealbumin (g/L)	278.3 ± 37.4	281.6 ± 39.8	0.590	211.6 ± 27.3	203.8 ± 23.1	0.044
Hemoglobin (g/L)	139.8 ± 13.6	141.2 ± 13.9	0.519	112.4 ± 9.6	110.7 ± 9.3	0.250
Meal size, *n* (g)	371.8 ± 67.3	367.5 ± 71.2	0.695	266.3 ± 40.2	253.6 ± 40.7	0.047
Meal times	2.3 ± 0.4	2.2 ± 0.5	0.176	3.1 ± 1.2	3.2 ± 0.9	0.528
Soft diet, *n* (%)	3 (4.9)	7 (5.7)	>0.999	32 (52.5)	59 (48.4)	0.601
Liquid diet, *n* (%)	28 (46.7)	62 (50.8)	0.530	25 (41.0)	56 (45.9)	0.528
Hard diet, *n* (%)	30 (49.2)	53 (43.4)	0.462	4 (6.5)	7 (5.7)	>0.999
Prognostic nutritional index	422.7 ± 75.3	437.02 ± 81.2	0.251	362.1 ± 61.4	369.5 ± 57.6	0.424
	Six months after surgery		* *	One year after surgery		* *
Body weight (kg)	58.2 ± 6.8	57.3 ± 6.3	0.376	58.6 ± 7.2	57.8 ± 6.9	0.467
Psoas muscle index (cm^2^/m^2^)	11.3 ± 2.4	10.6 ± 1.9	0.033	12.1 ± 1.4	11.9 ± 1.7	0.428
Serum albumin (g/L)	37.9 ± 3.6	38.3 ± 4.7	0.560	38.7 ± 5.1	38.9 ± 5.4	0.810
Serum prealbumin (g/L)	237.1 ± 24.6	232.4 ± 21.7	0.188	247.2 ± 31.3	244.3 ± 30.2	0.546
Hemoglobin (g/L)	114.7 ± 8.5	115.5 ± 8.1	0.536	118.2 ± 8.7	117.1 ± 7.4	0.373
Meal size, *n* (g)	279.4 ± 49.2	264.4 ± 45.2	0.041	311.5 ± 59.2	301.3 ± 62.4	0.291
Meal times	2.7 ± 0.8	2.8 ± 0.2	0.260	2.4 ± 0.2	2.5 ± 0.7	0.276
Soft diet, *n* (%)	21 (34.4)	39 (32.0)	0.738	10 (16.4)	18 (14.8)	0.772
Liquid diet, *n* (%)	31 (50.8)	68 (55.7)	0.529	35 (57.4)	77 (63.1)	0.453
Hard diet, *n* (%)	9 (14.8)	15 (12.3)	0.642	16 (26.2)	27 (22.1)	0.538
Prognostic nutritional index	370.4 ± 53.5	373.6 ± 55.8	0.711	379.5 ± 51.2	383.4 ± 51.4	0.629

*RG, robotic gastrectomy; LG, laparoscopic gastrectomy.*

**Table 7:** One year change rate of nutrition-related indexes after operation.

One year change rate	RG (*n* = 61)	PG (*n* = 122)	*P*
Body weight loss	−8.1 ± 1.7	−8.7 ± 1.9	0.039
Psoas muscle index	−10.9 ± 1.7	−11.4 ± 2.1	0.108
Serum albumin	−6.5 ± 0.7	−6.4 ± 0.6	0.317
Serum prealbumin	−5.7 ± 1.1	−5.9 ± 1.4	0.331
Hemoglobin	−7.4 ± 1.7	−7.2 ± 1.9	0.488
Meal size	−16.4 ± 3.9	−18.1 ± 4.3	0.010
Meal times	29.3 ± 6.5	31.2 ± 7.1	0.081
Prognostic nutritional index	−9.8 ± 2.6	−10.4 ± 3.2	0.206

*RG, robotic gastrectomy; LG, laparoscopic gastrectomy.*

**Table 8:** Correlation between nutritional status and quality of life in gastric cancer patients 1 year after surgery.

Categories	PG-SGA score			
	0–3	4–8	≥9	*P*
Physical symptoms				
Dysphagia	14.4 ± 6.1	15.2 ± 7.3	17.8 ± 9.2	<0.001
Sour regurgitation	7.2 ± 4.1	6.9 ± 3.4	7.7 ± 5.4	<0.001
Belching	8.4 ± 5.3	8.2 ± 4.6	8.9 ± 3.8	<0.001
Abdominal pain	16.3 ± 8.2	19.4 ± 7.7	22.6 ± 6.3	<0.001
Diarrhea	7.2 ± 5.2	9.3 ± 4.9	12.2 ± 6.4	<0.001
Fatigue	21.1 ± 9.3	22.5 ± 13.2	22.9 ± 12.6	<0.001
Anxious	6.3 ± 3.3	6.8 ± 4.1	7.4 ± 4.7	<0.001
Insomnia	23.6 ± 13.7	24.2 ± 12.9	23.9 ± 14.8	<0.001
Social function				
Independent living	92.2 ± 13.4	88.3 ± 16.2	82.4 ± 11.2	<0.001
Hobby	88.7 ± 12.3	84.2 ± 10.1	78.4 ± 9.8	<0.001
Exercise	89.3 ± 15.6	83.5 ± 11.2	80.1 ± 12.3	<0.001
Work efficiency	84.7 ± 14.4	80.3 ± 12.6	73.4 ± 10.7	<0.001
Quality of life score	94.7 ± 17.4	90.6 ± 15.9	83.9 ± 13.8	<0.001

## Discussion

A large number of studies have confirmed that robotic radical gastrectomy has the advantages of fewer complications, less bleeding, faster postoperative recovery, and shorter hospital stays compared with laparoscopic surgery (26–28). Moreover, robotic surgery can ensure that the surgical field is clean and clear, is easier to use for the surgeon and team, minimize the occurrence of vascular and organ side injuries, and increase the number of lymph nodes obtained. Lymph node dissection is the most complex and challenging part of radical gastrectomy. Some studies have shown that robots are more suitable for complex operations and lymph node dissection than laparoscopic radical gastrectomy for gastric cancer (29). This study shows that the average number of lymph nodes cleaned by robot surgery is 37.3, which is significantly higher than that of laparoscopic surgery is 32.8. This difference suggests that robot lymph node cleaning is superior to laparoscopic surgery, which may bring patients potential advantages in terms of better tumor treatment. This improvement may be related to the advantages of robot surgery, such as 10–15 times enlarged vision, distortion-free 3D display, and 7 degrees of freedom of surgical instruments, which make lymph node cleaning more accurate. Lee et al. (27) reported that patients with a high body mass index who underwent robotic-assisted distal gastrectomy plus D2 lymph node dissection had less blood loss and higher lymph node dissection quality. At the same time, since the operation time in this study does not include installation time, the operation time did not significantly differ between the two groups.

This study showed that the No. 1, No. 2, No. 3, and No. 7 lymph node metastasis rates were higher, followed by the metastasis rates of the No. 8, No. 9, No. 11, No. 19, No. 20, No. 110, and No. 111 lymph nodes; the lymph node metastasis rate of No. 4, No. 5, No. 6, and No. 12 were the lowest, which was similar to findings reported in the Japanese literature (26). These results all suggest that upper third gastric cancer is characterized by a unique pattern of lymph node metastasis, which can flow to the lower mediastinum and abdominal lymph nodes. Furthermore, the abdominal lymph nodes are the main lymph nodes, while the lower mediastinum still experiences a certain proportion of lymph node metastasis. Therefore, lymph node dissection for upper third gastric cancer should consider these two regions. The number of abdominal lymph nodes cleaned did not significantly differ between the robotic and laparoscopic groups, but the main difference lies in the subphrenic lymph nodes (No. 19 and No. 20) and the lower mediastinal lymph nodes (No. 110 and No. 111), and robot surgery is superior to laparoscopy.

In addition to the quality of radical surgery, the postoperative quality of life of gastric cancer patients is also the focus of surgeons (30). A number of clinical studies have confirmed the minimally invasive advantages of laparoscopic radical gastrectomy and its exact oncological efficacy. This study integrated the scales of the European Cancer Research and Treatment Assistance Organization (EORTC QLQ—C30 questionnaire and EORTC QLQ—STO22 questionnaire) to evaluate the quality of life recovery of patients in the robotic and laparoscopic groups after surgery. The results showed that dysphagia, abdominal pain, fatigue, diarrhea, and other physical symptoms after surgery were significantly better in the robotic group than in the laparoscopic group, which may be due to the more sophisticated operation of the robotic group (31). In addition, the amount of intraoperative bleeding was reduced in the robotic group, which may help reduce the formation of intra-abdominal adhesions and the resulting abdominal discomfort. These factors are conducive to the recovery of intestinal function after surgery.

The results of this study also showed that the social function scores of independent living, hobbies, fitness exercise, and work efficiency were better in the robotic group than in the laparoscopic group at 3–12 months after surgery, and the overall quality of life scores of the robotic group were better than those of the laparoscopic group. The reason may be that the robotic group has less intraoperative trauma, earlier bowel function recovery, an earlier return to a soft diet, a faster reduction of physical symptoms, less mental burden, and earlier physiological function recovery to the preoperative level (32). The results of this study also showed that the physical symptoms score of patients in the robotic group returned to the preoperative level at 6 months after surgery, and the improvement was more obvious at 12 months after surgery, while the physical symptoms score of patients in the laparoscopic group did not return to the preoperative level until 12 months after surgery. Therefore, compared with the laparoscopic group, patients in the robotic group were able to achieve self-care earlier, were more willing to resume leisure activities and fitness exercises, had higher work efficiency, and were better able to recover their social roles.

Due to the changes in the anatomical and physiological structure of the digestive tract and the impairment of gastrointestinal function after operation, patients undergoing radical gastrectomy are recommended to eat smaller and more frequents meals, mainly a liquid diet (33). However, this study found that more patients in the robotic group ate a solid diet and soft food than those in the laparoscopic group at 3, 6, and 12 months after surgery and had a greater tendency to recover to the proportion of preoperative dietary components. This finding indicated that patients in the robotic group were more able to tolerate a solid diet after surgery. Furthermore, the robotic group had a lower score of dysphagia, diarrhea, and other physical symptoms than the laparoscopic group, which further indicated that gastrointestinal symptoms in the robotic group recovered quickly in the early postoperative period.

The recovery of postoperative nutritional indicators is also an important standard to consider the quality of life of gastric cancer patients after surgery. Lower changes in nutritional indicators and faster nutritional recovery after surgery tend to promote a better prognosis, indicating better postoperative quality of life (34). The results of this study showed that the proportion of changes in body weight and meal size were significantly lower in the robotic group than in the laparoscopic group 1 year after surgery. This difference may be related to the fact that the patients in the robotic group have a higher tolerance to diet than those in the laparoscopic group and can quickly recover their preoperative eating habits after surgery. In addition, the patients in the robotic group consumed a solid diet as early as possible after the operation, which provided them with more energy and nutrients needed by the human body, thus promoting the maintenance of body weight after the operation.

We found that as the PG-SGA score increased, values from the functional category and for the overall health status of patients with a lower mean field rank and the symptoms category rank mean increased. Specifically, as functional abilities and quality of life worsened, symptoms or problems, such as fatigue, nausea, belching, diarrhea, and insomnia, worsened and added to the poor quality of life.

### Learning curve

Although studies regarding the learning curve of robotic gastrectomy are scarce, all reported that the robotic system is more adaptable than the laparoscopic environment (35–37). Moreover, in contrast to the longer operation time, the robotic system makes surgeons rapidly overcome the learning curve for robotic gastrectomy, which may help less-experienced surgeons. The actual impact of the robotic system on the learning curve of robotic gastrectomy is difficult to evaluate without considering the experience of laparoscopic gastrectomy because the robotic gastrectomy procedure is identical to laparoscopic gastrectomy. Thus, the exact assessment of the learning curve effect would be difficult.

### Cost

Studies have consistently reported that the costs of robotic gastrectomy are higher than those of laparoscopic gastrectomy. Robotic gastrectomy consistently costs more than laparoscopic gastrectomy. The high cost of robotic gastrectomy is mainly associated with the cost of robotic system installation and disposable drapes and instruments (38, 39). Moreover, since a longer operation time itself means another source of extra expense of robotic surgery, balancing the cost of robotic surgery with that of laparoscopic surgery is difficult. Thus, further studies to determine whether the benefits of robotic surgery would reduce the other costs related to postoperative care or readmission are necessary.

This research is subject to limitations. The malnourished patients did not receive further nutritional intervention, and we hope to clarify in future research whether an improvement in the nutritional status in gastric cancer patients will improve clinical outcome. In addition, the effect of nutritional status on the final clinical outcome after nutritional therapy was not followed up.

## Conclusion

In summary, robotic-assisted radical total gastrectomy for upper third gastric cancer is safe and feasible. Compared with laparoscopic surgery, it is more sophisticated, has less bleeding, and has a higher quality of lymph node dissection, especially for subphrenic and lower mediastinal lymph nodes. At the same time, patients in the robotic group also had better quality of life and faster postoperative nutritional recovery than patients in the laparoscopic group.

## Data Availability

The original contributions presented in the study are included in the article/supplementary material, further inquiries can be directed to the corresponding author.
